# The Foes of Hospital Economy

**Published:** 1920-11-13

**Authors:** F. A. Hocking

**Affiliations:** London Hospital, Whitechapel, E.


*To the Editor of* The Hospital.

### "The Foes of Hospital Economy."

*To the Editor of* The Hospital.

Sir,—I have been reading your comments on Mr. Dunstan's paper advocating co-operation and standardisation in hospital buying. Some eleven or twelve years ago I published a detailed article on almost exactly the lines he now suggests, hence it might be supposed I should endorse his sentiments. Much water has, however, passed under London Bridge since then, and the experience of the last few years, particularly of the last five, has made me very sceptical as to any advantage to be gained in the direction advocated. Mr. Dunstan admits that "many of the larger places in London probably would not benefit to any great extent." Any benefit would accrue to the smaller institutions, "which would save large sums of money." Would they? In many instances, if the *whole drug and dressing bill* were abolished the saving effected could not be described as "large."

Reform in the direction of Dispensary supplies may be desirable, but I venture to suggest that such reform should proceed on the following lines:—

1. The dispensary of each hospital should be under the charge of a pharmacist—*i.e.*, one who possesses the qualifying certificate of the Pharmaceutical Society.

2. That each pharmacist should be held responsible for the efficiency of his department, and to that end he should be allowed all reasonable freedom in carrying on his work.

3. That before any costly experiments in co-operative buying be undertaken, some concerted effort be made on the part of small and medium-sized hospitals to obtain and exercise certain knowledge—viz., that of the proper sources of supply. My meaning may be illustrated by the following incident.

A pharmacist of a provincial hospital on a visit to London for the purpose of obtaining supplies showed me a list of his requirements. I was surprised to note therein the name of an article which is manufactured in his own county, and only a few miles from his institution. Yet he had come to London to buy it!

It should have been quite within his abilities to have made all his purchases, expending a shilling or two on postages, on terms at least equal to those he secured in London, without the added expense of a journey to the Metropolis.

As regards his remark about manufacturing "scores of items now bought from the trade," I merely say that hospital pharmacists of my acquaintance have quite enough to do in discharging their legitimate duties, without indulging in the extravagant luxury of setting up a miniature Crosse and Blackwell factory.

In conclusion, the truth respecting co-operative buying of drugs and dressings cannot be elicited by means of magazine articles and correspondence. Why not set up a representative committee, including in its membership two or three pharmacists of our big hospitals, to inquire into and report upon the subject.—Yours truly,

London Hospital, Whitechapel, E. F. A. Hocking.

